# Sleep-inducing effect of Rhynchophylline in *EphA4* knockout mice

**DOI:** 10.1093/sleepadvances/zpad037

**Published:** 2023-09-27

**Authors:** Maria Neus Ballester Roig, Valérie Mongrain

**Affiliations:** Department of Neuroscience, Université de Montréal, Montréal, QC, Canada; Center for Advanced Research in Sleep Medicine, Recherche CIUSSS-NIM, Montréal, QC, Canada; Department of Neuroscience, Université de Montréal, Montréal, QC, Canada; Center for Advanced Research in Sleep Medicine, Recherche CIUSSS-NIM, Montréal, QC, Canada; Centre de Recherche du Centre Hospitalier de l’Université de Montréal (CRCHUM), Montreal, QC, Canada

**Keywords:** ephrin type-A receptor 4, slow wave sleep, paradoxical sleep, electrocorticographic activity, mouse

## Abstract

We have recently demonstrated that the alkaloid rhynchophylline (RHY; purified from *Uncaria* plants) induces sleep and modifies electrocorticographic (ECoG) activity throughout the 24-h day in a vigilance state-dependent manner in wild-type mice. We here asked whether this alkaloid impacts wake/sleep variables in the absence of the cell adhesion protein EPHA4, via ECoG recording in *EphA4* knockout (KO) mice submitted to the same RHY treatment contemporaneously to the wild-type mice (littermates). We uncover that RHY decreases time spent awake and increases time spent in slow wave sleep in *EphA4* KO mice and alters the 24-h time course of ECoG activity during wakefulness and sleep states. These observations are similar to the reported effects of RHY in wild-type littermate animals, which strongly supports that RHY-driven sleep alterations are not dependent on the presence of EPHA4.

**Statement of significance**

The findings show that the alkaloid rhynchophylline increases time spent in slow wave sleep and alters synchronized brain activity in comparison to the control condition saline in mice lacking the EPHA4 cell adhesion molecule (*EphA4* KO mice). This observation supports that the effect of this alkaloid on sleep/wake phenotypes are independent of EPHA4.

Rhynchophylline (RHY) is one of the main alkaloid components of the plant *Uncaria rhynchophylla* that is used in traditional Asian medicine notably for having sleep-inducing properties [1, 2]. In mice, we specifically showed that RHY increases slow wave sleep (SWS) when administered in the early light period and immediately before the dark period [3]. In the context of animal models of neurodegenerative and psychiatric diseases, some studies have suggested that RHY acts on the central nervous system by preventing the activation (via phosphorylation) of the ephrin receptor EPHA4 as well as by preventing the activation of EPHA4 downstream pathways (e.g. CDK5 phosphorylation) [4, 5]. In addition, one of these studies showed that RHY was able to physically bind to EPHA4 using a pull-down assay [4]. However, a direct RHY and EPHA4 interaction is not supported by findings using nuclear magnetic resonance and isothermal titration calorimetry assays [6], which could question whether EPHA4 is targeted by RHY. EPHA4, considered a cell adhesion protein and tyrosine kinase, was shown to have a role in central nervous system development and to regulate the morphology of dendritic spines [7, 8], and our previous work indicated that *EphA4* knockout (KO) mice have alterations in sleep [9, 10]. We, therefore, hypothesized that RHY modifies sleep by acting on the EPHA4 receptor, and tested the specific assumption that RHY would fail to induce sleep in *EphA4* KO mice.

*EphA4* KO mice [7] were originally kindly provided by Keith K. Murai (McGill University). Animals were backcrossed for >10 generations to C57BL/6J mice and then bred on site. Male and female mice (*EphA4* KO and WT littermates) followed the electrocorticographic (ECoG)/electromyographic (EMG) electrode implantation and experimental conditions described in our recent study [3]. WT littermates and KO mice have been instrumented and recorded simultaneously (i.e. over the same experimental months), but only data of WT mice have been reported recently [3]. Briefly, after recovery and habituation, ECoG/EMG signals were recorded for a 24-h injection (INJ) day during which animals were injected intraperitoneally at ZT0 and ZT11 with saline or 100 mg/kg of RHY (Baoji Herbest Bio-Tech Co. Ltd, #76-66-4, diluted in 0.9% NaCl). More precisely, four KO females received saline (11.8 ± 0.6 weeks old, 16.7 ± 1.0 g), five KO males received saline (12.2 ± 1.3 weeks old, 21.8 ± 2.3 g), five KO females received RHY (12.2 ± 1.4 weeks old, 18.6 ± 2.1 g), and six KO males received RHY (12.0 ± 1.1 weeks old, 22.4 ± 0.9 g). RHY was reported to cross the blood–brain barrier [11, 12], and the selected dose of 100 mg/kg (RHY100) was specifically shown to decrease time spent in wakefulness and to increase time spent in SWS in WT mice, as well as to have larger effects on wake/sleep variables in comparison to a 50 mg/kg dose [3]. The visual identification of wakefulness, SWS, and paradoxical sleep (PS) was done on a 4-s epochs using ECoG/EMG traces by the first author who was blind to RHY treatment and sex. The INJ day was then analyzed for percent time spent in wakefulness, SWS, and PS during the full 24 h, the distribution of time spent in these states over the 24 h, the mean duration of individual bouts of the three states during the full 24 h, and the 24-h distribution of the number of individual bouts. In addition, the 24-h time course of wake alpha (8–12 Hz) activity, SWS delta (1–4 Hz) activity, SWS theta (6–9 Hz) activity, and SWS sigma (10–13 Hz) activity of a ECoG frontal–parietal bipolar signal was compared between saline and RHY treatments using intervals to which an equal number of wake or SWS epochs contributed as done previously [3, 9]. PS theta activity was analyzed per 6-h intervals to ensure a sufficient number of artifact-free epochs contributing to datapoints for each mouse. One male from the saline group was excluded for all ECoG activity analyses because of low-signal quality, and one female from the saline group was additionally excluded for the analysis of theta activity per 6-h intervals because of an absence of PS for one interval.

RHY was found to significantly reduce the percent time spent in wakefulness by more than 20% relative to saline and to significantly increase the percent time spent in SWS by more than 30% relative to saline for the full 24-h INJ day (combined females and males saline 57.0% vs. females and males RHY with 43.1% of recording time spent awake; combined sexes saline 39.1% vs. RHY 52.1% recording time spent in SWS; Figure 1A). When considering the 24-h distribution of states (Figure 1B), RHY was found to significantly decrease time spent awake during the middle of the light period and more strongly during the first half of the dark period, and to significantly increase time spent in SWS during the early light and dark periods. Moreover, RHY reduced time spent in PS by more than half for the second to the fourth hours of the light period, but increased by more than twofold the time spent in PS between hours 2 to 7 of the early dark period. These changes were accompanied by indications of higher wake/sleep fragmentation under RHY. Indeed, RHY significantly decreased the duration of individual wake and SWS bouts (wake bout duration about 50 s shorter and SWS bout duration about 11 s shorter; Figure 1C), and increased the number of wake and SWS bouts mainly in the first hours following injections (Figure 1D). PS bout number was decreased following early day injection but increased after late day injection. It should be noted that the 24-h distribution of the number of bouts is nearly identical for wake and SWS given the high predominance of wake-SWS transitions due to the minor contribution of PS (Figure 1D).

**Figure 1.**
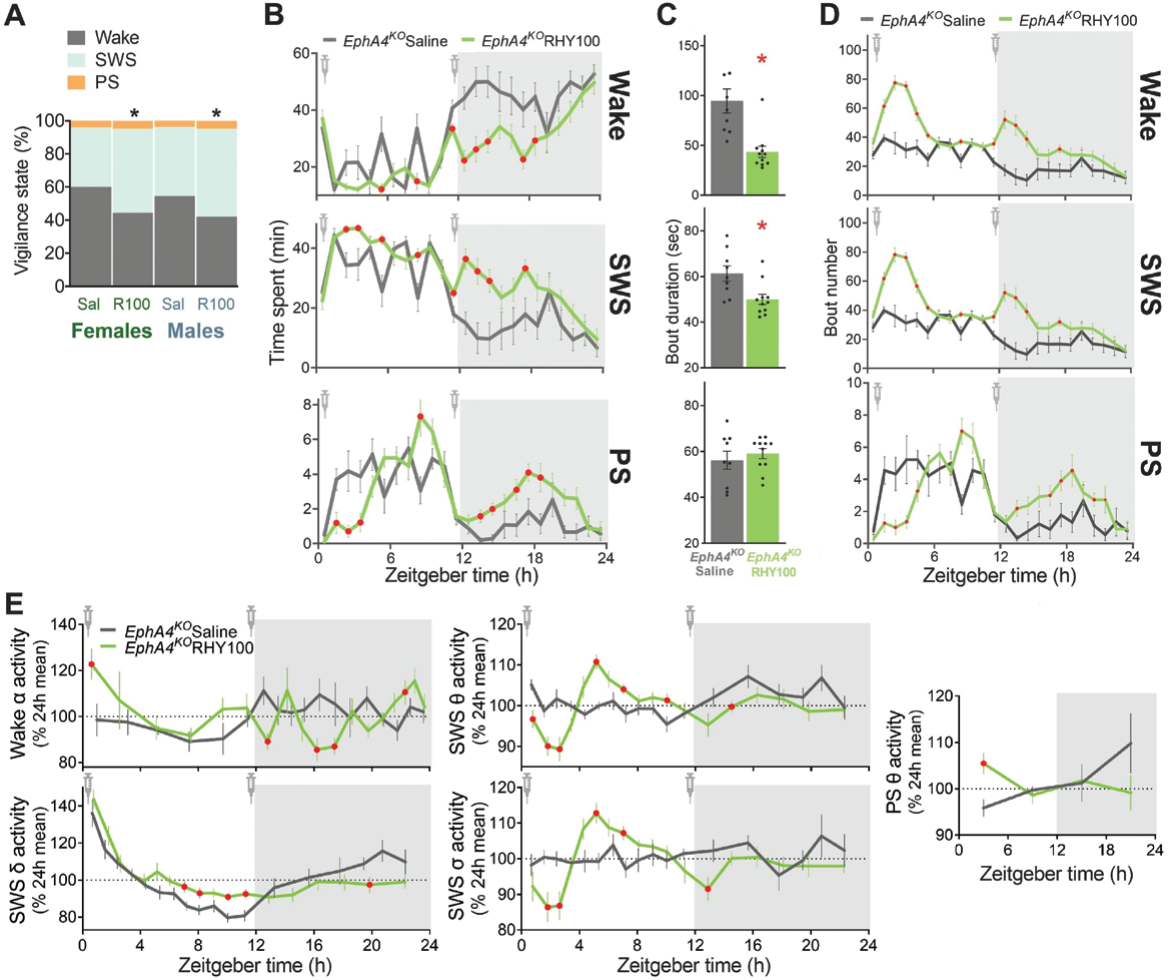
RHY induces SWS and modifies ECoG activity in *EphA4* KO mice. (A) Percent time spent in wakefulness, SWS, and PS for the full 24-h recording was compared between males and females and between saline and RHY-injected KO mice using factorial analyses of variance (ANOVAs). Main treatment effects were found for wake, SWS, and PS (F_1,16_ > 4.5, *p* < .05; collectively shown by stars), indicating significant differences between saline and RHY treatments. No main sex effect or treatment by sex interaction was found (main sex effect F_1,16_ < 1.5, *p* > .2; interaction F_1,16_ < 0.3, *p* > .6). (B) The hourly distribution of wake, SWS, and PS over 24 h was compared between saline and RHY treatments using repeated-measure ANOVAs (males and females combined; also used in D and E). Significant treatment by hour interactions were found for all three states (F_23,414_ > 2.9, Huynh-Feldt corrected *p* < .01). Red datapoints indicate significant differences from the saline group (planned comparison *p* < .05; also in panels D and E). Zeitgeber time 0 indicates lights ON and Zeitgeber time 12 lights OFF, with gray areas representing the dark period (also in panels D and E). (C) The mean duration of individual bouts of wakefulness and SWS calculated for the full 24-h recording was significantly decreased by RHY in comparison to saline (*t* > 2.9, *p* < .01), but not the mean duration of individual PS bouts (*t* = −0.7, *p* = .5). (D) The hourly distribution of the number of bouts over 24 h was compared separately for the three states. Treatment by hour interactions were significant for wake, SWS, and PS (F_23,414_ > 4.9, Huynh-Feldt corrected *p* < .001). (E) The time course of wake alpha (α: 8–12 Hz), SWS delta (δ: 1–4 Hz), SWS theta (θ: 6–9 Hz), SWS sigma (σ: 10–13 Hz), and PS theta (θ: 6–9 Hz) activity was compared between saline and RHY. Significant treatment by interval interactions were found for all five dynamics (F_3/17,48/272/289_ > 2.0, Huynh-Feldt corrected *p* < .05).

In parallel, RHY significantly modified the 24-h time course of wake and sleep ECoG activity in *EphA4* KO mice (Figure 1E). More precisely, wake alpha activity was increased by RHY by more than 20% for the first interval of the light period and decreased by more than 10% for some intervals during the first half of the dark period. SWS delta activity was increased by RHY in the second half of the light period and decreased during the late dark period. RHY altered the time course of SWS theta and sigma activity similarly; with decreases (of about 10 %) in the early light period and increases (of 10 % or less) in the mid-light period. RHY also increased theta activity during PS specifically during the first 6 h of the light period. Therefore, RHY considerably modified ECoG activity dynamics during wakefulness and sleep states in *EphA4* KO mice.

Overall, RHY was found to have wake-suppressing and SWS-promoting effects in *EphA4* KO mice. This was observed for both the light and dark periods, with an apparent stronger effect when animals are usually awake (i.e. at the beginning of the dark period) and occurred together with an increased frequency of shorter wake/SWS bouts. These findings are strikingly similar to those reported in WT mice from the same litters [3]. Of great similarity with observations in WT is also the PS-suppressing and PS-inducing effects of RHY in the light and dark periods, respectively, alterations in the 24-h dynamics of wake alpha, SWS delta, and SWS sigma activity, as well as the increase in PS theta activity. Our findings thus support that the effects of RHY on wake/sleep amount and architecture in mice are not mediated by EPHA4 and support that RHY is globally sleep-promoting.

Our recent study showed an absence of effect of RHY on EPHA4 protein level and phosphorylation in WT mice [3], which also provides support to the idea that sleep-promoting effects of RHY are independent of EPHA4. This is in line with empirical observations of a lack of effect of RHY on EPHA4-EFNA1 binding, and an absence of direct RHY-EPHA4 interaction found with nuclear magnetic resonance and isothermal titration calorimetry assays [6, 13] but not with findings of direct physical interaction between RHY and EPHA4 reported using a pull-down assay [4]. However, it remains possible that developmental compensation related to the absence of EPHA4 could have masked its contribution to RHY-mediated wake/sleep changes. EPHA4 is reported to be involved in neurodevelopment, including in corticogenesis and the organization of thalamocortical circuits [7, 14, 15]. Also, we have reported an increased gene expression of *EfnB2* (coding for Ephrin-B2, a physical partner of EPHA4 and other EPH receptors) in the forebrain of *EphA4* KO mice [9], and an increased *EphA5* cortical expression was also reported in these mice [14], which could both suggest some compensation by other cell adhesion proteins of the Ephrin-EPH system. Of note is also that we found forebrain genome-wide gene expression to be preserved in *EphA4* KO mice, including when assessed after sleep deprivation [9], a finding that might support some level of compensatory signaling in these mice. In addition, under disease model/pathological conditions, RHY may still modulate EPHA4 phosphorylation via indirect mechanisms in a manner affecting neuronal function [4, 5]. To conclude, our results suggest that EPHA4 is not a main contributor of the sleep-inducing properties of RHY, which likely shapes the distribution of wake/sleep states and ECoG activity by acting on multiple molecular routes including the hypocretin/orexin and melanin-concentrating hormone systems [3].

## Data Availability

The data underlying this article will be shared on reasonable request to the corresponding author.
